# Fractionation-Based Modeling of Hawthorn (*Crataegus* L.) Preparation Bioactivity: Relationship Between Fraction Composition and Mechanisms of Action in Colon Cells

**DOI:** 10.3390/molecules31152694

**Published:** 2026-08-03

**Authors:** Natalia Żurek, Ireneusz Kapusta

**Affiliations:** Department Food Technology and Human Nutrition, Faculty of Technology and Life Science, University of Rzeszów, 4 Zelwerowicza St., 35-601 Rzeszów, Poland

**Keywords:** seeds, hawthorn, proanthocyanidins, UPLC, large intestine

## Abstract

Oxidative stress and inflammation are the causes of many colonic diseases. Therefore, the aim of this study was to maximize the biological activity of hawthorn seed preparations aimed at modulating oxidative stress and the inflammatory response in the colon through selective fractionation and enrichment in compounds with antioxidant, anti-inflammatory, and cytotoxic potential. Fractionation of hawthorn seed extract (CE) was performed using C18 resin, and detailed phytochemical analysis was performed using ultra-performance liquid chromatography (UPLC-MS/MS). This work resulted in four fractions (F1-F4), with differential distribution of 28 identified polyphenolic compounds. Phenolic acids dominated in F1, flavan-3-ols in F2, and flavonols in F3 and F4. In terms of quantitative composition, the obtained fractions can be ranked in the order F1 > F2 > F3 > F4. In biological activity studies, the highest antioxidant activity in a chemical model was demonstrated for F2, which was also confirmed in a cellular model–colonocyte cells (CCD841CoN line) stimulated with H_2_O_2_. F2 also demonstrated the highest inhibition of ROS production by colonocytes and NO production by macrophages. High F2 activity was also demonstrated in studies of the proliferation, migration, and invasion of colon cancer cells. These findings underscore the validity of fractionation of hawthorn seed extract and its potential use in the prevention and treatment of colon diseases. Future studies should include in vivo models and estimation of the activity of the fraction truly bioavailable after digestion.

## 1. Introduction

It has long been known that oxidative stress plays an important role in the pathogenesis of colon diseases, including inflammatory diseases and colon cancer [1]. Under conditions of chronic oxidative stress, the overproduction of reactive oxygen species (ROS) and nitrogen species (RNS) leads to DNA, lipid and protein damage, mutations and activation of signaling pathways such as NF-κB or MAPK, promoting the progression of inflammation and neoplastic transformation [2].

In the above context, polyphenolic compounds isolated from plant matrices are the subject of intensive scientific research and a potential tool in the prevention of diseases dependent on oxidative stress [3]. At the same time, agricultural waste can be a sustainable and cheap source of bioactive compounds with an important role in the food and pharmaceutical industries [4,5]. One such waste product is hawthorn seeds (*Crataegus* L.). Hawthorn berries are a widely known pharmaceutical raw material for the production of extracts used in the treatment of cardiovascular diseases, as well as a food raw material for the production of tinctures, jams, and functional juices [6,7]. A byproduct of these processes is the seeds, a raw material that has been largely unexplored and underutilized. In our previous report, we conducted a phytochemical analysis of the seeds of various hawthorn species for the first time [8]. In the cited work, we identified 23 polyphenolic compounds, and demonstrated antidiabetic, antiobesity, anti-inflammatory, and cytotoxic effects on cancer cells. Of the six species tested, the highest mentioned properties were demonstrated for *C. macrocarpa*. In other studies, hawthorn seeds have also been shown to have antioxidant, antibacterial [9,10], and anti-inflammatory and neuroprotective activity [11].

As can be observed, hawthorn seeds are an example of a plant matrix with high nutraceutical potential and potential applications in functional products. However, we hypothesize that maximizing their functional properties will be achieved after fractionation of polyphenolic compounds [12]. Obtaining preparations with a high content of specific polyphenols in a small mass of the preparation may allow for increased bioavailability and biological effectiveness [13]. Due to the growing interest in the search for natural strategies to support health, the above approach to obtaining bioactive preparations is becoming increasingly important, which is also beneficial from an economic and environmental point of view.

Therefore, the aim of this study was to verify the use of C18 fractionation to obtain preparations rich in specific groups of polyphenolic compounds and its effect on the level of antioxidant, anti-inflammatory, and anticancer activity in an in vitro model of the colon. It was assumed that it would be possible to select the solvent concentration to increase selectivity and obtain preparations with increased concentrations of the desired compounds. The polyphenol profile of the preparations was analyzed using ultra-performance liquid chromatography coupled with mass spectrometry (UPLC-MS/MS). Furthermore, the assessment of antioxidant and anti-inflammatory activity was conducted in a chemical and cellular model using human colonocytes. The ability of the obtained preparations to inhibit the colonization, migration, and invasion of colon cancer cells was also assessed. This report is the first comprehensive report on the correlation between specific groups of polyphenolic compounds and the colon-protective properties of fractionated hawthorn seed extracts targeted to the colon.

## 2. Results and Discussion

### 2.1. Content of Polyphenolic Compounds in Fractions

**Figure 1:**
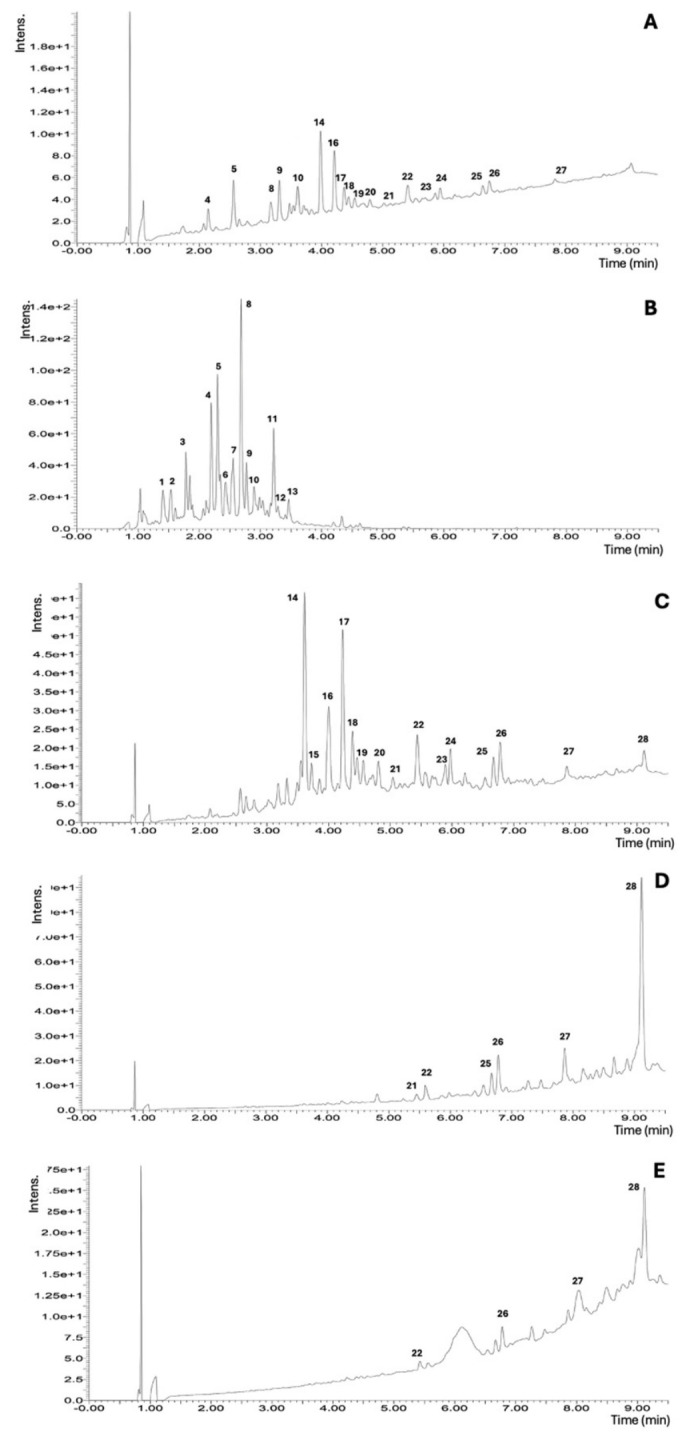
Images of UPLC chromatograms of crude extract (**A**) and polyphenolic fractions F1, F2, F3 and F4 (**B**–**E**, respectively) isolated from hawthorn seeds. Peak numbering according to Table S1.

The qualitative and quantitative composition of polyphenolic compounds in the CE and the obtained fractions is presented in Table 1, Figure 1 and Table S1. Fractionation on a C18 column allowed for obtaining preparations with very specific, diverse polyphenolic profiles, concentrated in a small mass of the preparation. A total of 28 polyphenolic compounds were identified, of which 19 compounds were found in CE, while 13, 15, 6 and 4 were identified in F1, F2, F3 and F4, respectively. As can be seen in Figure 1, the distribution of peaks was consistent with the elution time of the polyphenolic compounds. This is a typical phenomenon resulting from differences in the affinity of individual compounds for the stationary phase and their polarity, simultaneously allowing for the selective separation and isolation of specific classes of polyphenolic compounds [12].

The polyphenolic composition of CE included phenolic acids (46.7%) and flavan-3-ols (43.9%) in comparable amounts. Of these two classes of compounds, coumarylquinic acid isomer II (3.1 mg/g) and (+)catechin (3.2 mg/g) were found in the highest concentrations. Flavonols accounted for 9.5% of the total polyphenolic compounds. The total polyphenol content in CE was 18.7 mg/g. This value is 6.0 and 4.4 times higher compared to the content of polyphenols in blackberry seeds [14] and sea buckthorn seeds [15], respectively, and 3.1 and 3.2 times lower compared to black raspberry seeds [16] and grapes [17], respectively. This varied polyphenol content is primarily due to the genetic makeup of a given species, as well as environmental conditions, the stage of fruit ripeness and the extraction method [18]. F1 was composed primarily of phenolic acids (88.4%), with the highest concentrations of coumarylquinic acid isomer I (38.9 mg/g) and 5-*O*-caffeoylquinic acid (30.1 mg/g). Phenolic acids are a typical group of compounds found in berry seeds. They dominate in strawberry and raspberry seeds, among others [18]. At the same time, the highest total amount of polyphenols was identified in F1 (154.9 mg/g), of which 11.6% of the composition was also flavan-3-ols. In F2, flavan-3-ols dominated (91.3%), represented mainly by (+)catechin (28.3 mg/g) and procyanidin tetramer (23.6 mg/g). Other groups of compounds present in this fraction included phenolic acids (12.7%) and flavonols (5.7%). Proanthocyanidins are considered the most valuable class of polyphenolic compounds present in the seeds [19,20]. Their content may range from 0.9 mg/g (raspberry seeds) [21] to 8.1 mg/g (grape seeds) [22]. In relation to the pathogenesis of colon diseases, it has been shown that the delivery of proanthocyanidins in an unchanged form to this part of the digestive tract has a beneficial effect on the intestinal microbiota, as well as a direct biological effect on the intestinal epithelial cells [23]. F3 was mostly composed of flavonols (98.1%), with the remainder being phenolic acids. The dominant compound in this group was an unspecified quercetin derivative (55.1 mg/g). In addition to quercetin derivatives, kaempferol derivatives were also present. These are the flavonols that generally dominate in berry seeds [22]. To a lesser extent, isorhamnetin, rutin and luteolin are also identified [14,16,17,21]. In the last fraction, F4, only flavonols were identified, where unspecified quercetin derivative also dominated (2.0 mg/g), with a total polyphenol content of 3.7 mg/g.

### 2.2. Antioxidant Activity of Fractions in the Chemical Model

The chemical antioxidant activity of polyphenolic compounds depends on their chemical structure, concentration, and solubility, but also on several external factors, such as time, pH and temperature of the reaction environment [24]. Therefore, five methods, each based on different mechanisms of action, were used to assess antioxidant activity in the chemical model. We believe that this methodology ensures a reliable assessment of the use of the obtained preparations as a source of antioxidants.

**Figure 2:**
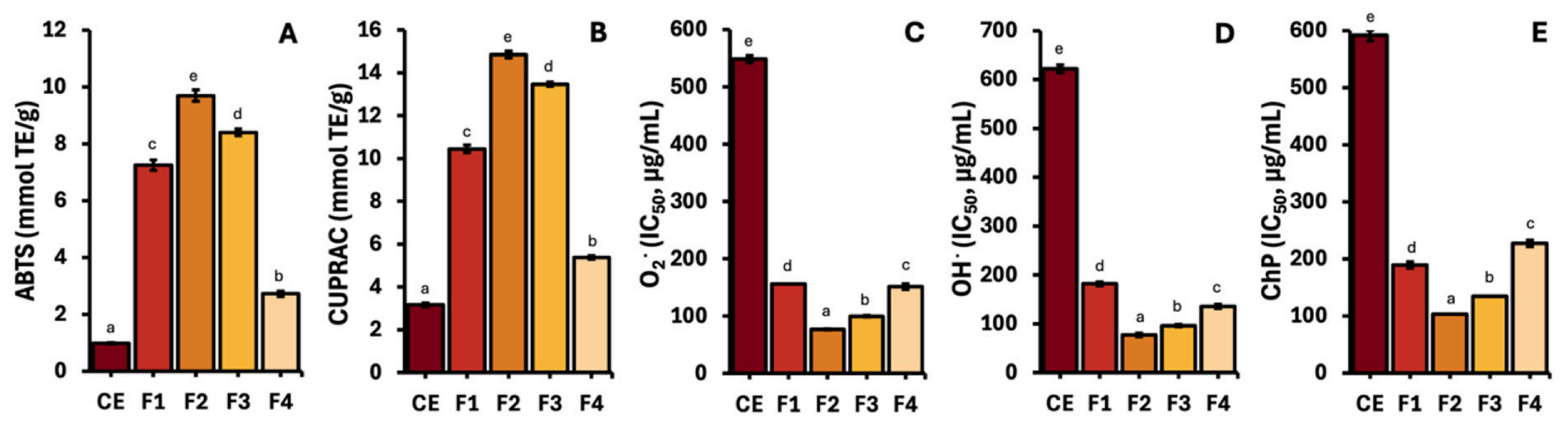
Antioxidant activity of crude extract (CE) and polyphenolic fractions (F1, F2, F3, F4) isolated from hawthorn seeds, assessed by the ABTS test (**A**), CUPRAC test (**B**), O_2_^•−^ radical scavenging test (**C**), OH^•^ radical scavenging test (**D**) and metal ion chelating capacity (**E**). Statistically significant differences between samples are marked with different letters (a–e) for *p* < 0.05.

In each of the tests performed, the highest antioxidant activity was demonstrated for F2. For the ABTS and CUPRAC tests, these values were 9.7 and 14.9 mmol TE/g, respectively, while in the O_2_^•−^, OH^•^ and ChP tests, these were 76.8, 77.2 and 103.6 µg/mL (Figure 2). Statistically significant differences between the analyzed fractions can be explained by their qualitative composition. Proanthocyanidins, which dominate in F2, are generally strong antioxidants [25]. This is due to the presence of numerous hydroxyl groups in the aromatic rings, the specific stoichiometry of their molecular structure and the synergistic interaction between the polymers [26]. In previous studies of rosehip extract, despite the overall low sum of all polyphenolic compounds compared to other extracts analyzed in this study, 98% of its composition were flavan-3-ols, which allowed for obtaining the highest antioxidant activity [27]. In the cited study, lower activity was demonstrated for marigold extract, despite its higher total polyphenol content, which was composed of 95% flavonoids. This group of compounds was identified in the highest concentration in F3. Zeng et al. [28] compared the antioxidant activity of flavonoids in the ABTS and DPPH tests and ranked their antioxidant power in the following order: procyanidin B2 > epigallocatechin > quercetin > epicatechin > rutin. It has been reported that the presence of a sugar residue in the ring of quercetin and kaempferol derivatives is the main factor reducing their antioxidant activity in the chemical model [29]. This may explain the lower activity of F3 compared to F2. In turn, among the analyzed fractions, F4 and F1 showed the lowest antioxidant activity in the chemical model. F4 was characterized by an overall low polyphenol content, which could have translated into the obtained results. However, F1 was dominated by phenolic acids, which, due to their simple structure and the presence of one or two hydroxyl groups, have a lower potential for scavenging synthetic radicals compared to more complex polyphenols such as proanthocyanidins [30]. Previously, in one of the studies, scientists also proved that an important factor influencing the antioxidant effect of phenolic acids is their concentration [24]. At low doses (100 µM), phenolic acid combinations demonstrated stronger antioxidant properties than at higher concentrations (1000 µM). In the case of CE, the lower content of polyphenolic compounds, as well as the presence of additional substances in the extract, such as fiber, protein, and pectin, could have resulted in limited antioxidant release from the plant matrix and overall lower antioxidant activity. This was previously confirmed in the studies of Lachowicz-Wiśniewska et al. [31] for chokeberry fruit extracts, also emphasizing the influence of the above-mentioned limiting factors on the low stability and bioavailability during digestion of polyene compounds from the matrix of crude extracts compared to purified extracts.

The above findings highlight the complex interactions and concentration dependencies between polyphenolic compounds in shaping antioxidant activity in the chemical model.

### 2.3. Antioxidant and Anti-Inflammatory Activity of Fractions in a Cellular Model

Monitoring plant materials for their antioxidant activity in a cellular model is crucial due to their important role in the human body. This allows for the assessment of potential protective properties against cellular damage caused by oxidative stress. Such assessment also allows for the verification of the nutraceutical potential of preparations for use in the prevention and treatment of diseases related to oxidative disorders [32]. Therefore, in our study, we assessed the effect of seed preparations on inhibiting oxidative damage to colonocytes (CCD841CoN) induced by H_2_O_2_. For the experiments, we selected a concentration of 0.125 mM H_2_O_2_ (Figure S1) and biocompatible concentrations of seed preparations.

**Figure 3:**
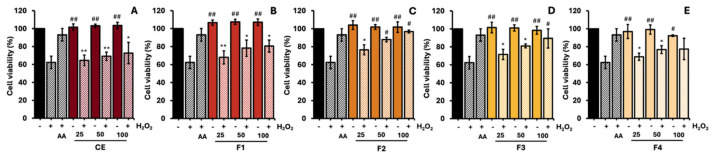
Effect of crude extract (CE; **A**) and polyphenolic fractions (F1, F2, F3, F4; **B**–**E**, respectively) from hawthorn seeds on the viability of human colonocytes (CCD841 CoN) treated with H_2_O_2_. Ascorbic acid (AA) at a concentration of 5.0 μg/mL. Statistically significant differences compared to the control are marked with *, # and **, ## for *p* < 0.05 and *p* < 0.01, respectively. * denotes a statistically significant difference in comparison to control cells not treated with H_2_O_2_; # compared to control cells treated with H_2_O_2_.

The results show that antioxidant activity differed between the tested preparations (Figure 3). The highest ability to mitigate H_2_O_2_-induced damage was demonstrated for F2. Colonocyte viability after H_2_O_2_ treatment and F2 administration at the highest dose tested (100 μg/mL) was 96.74%, which was clearly better compared to H_2_O_2_ treatment alone (62.33%) and the positive control (ascorbic acid, 93.12%).

**Figure 4:**
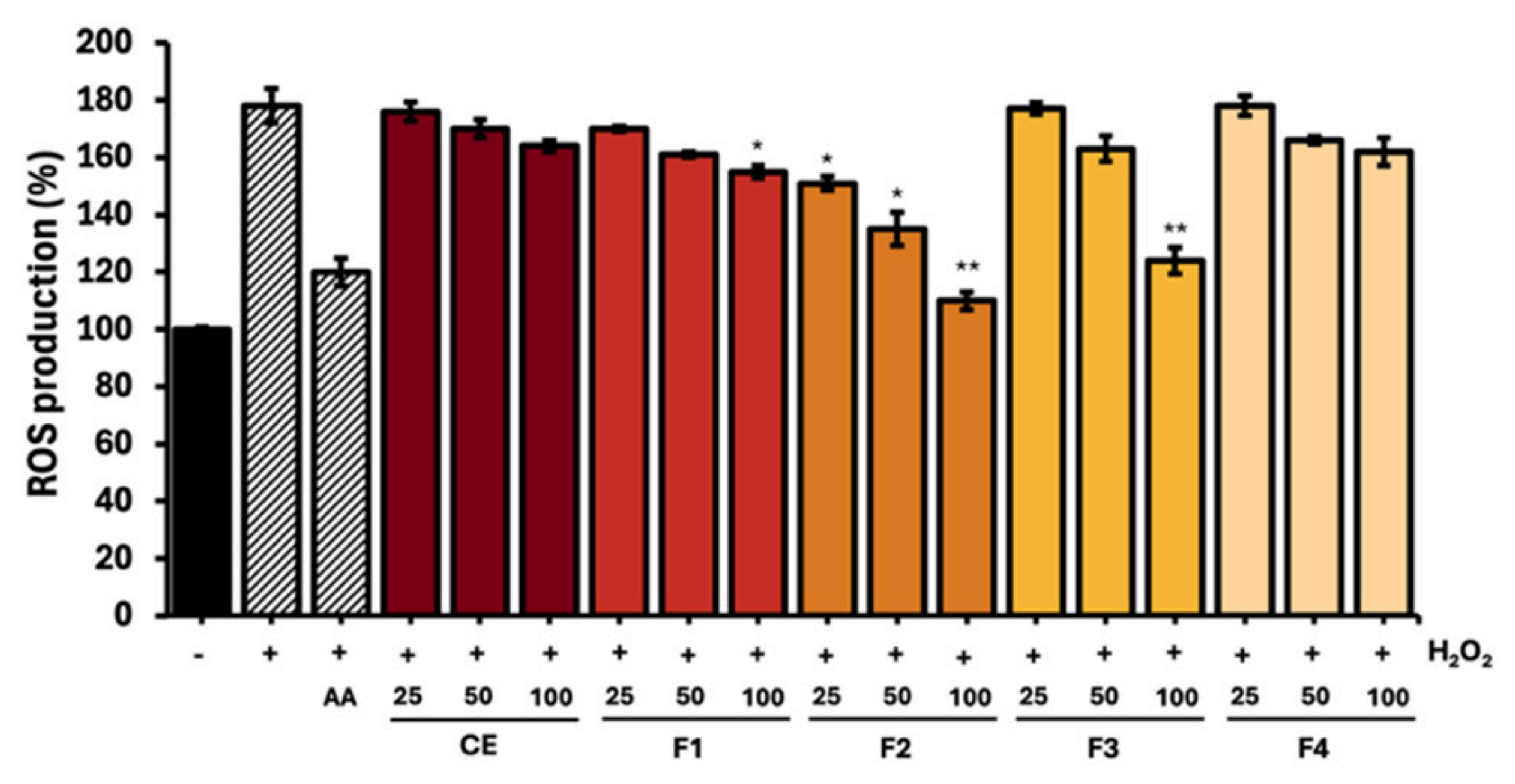
Effect of crude extract (CE) and polyphenolic fractions (F1, F2, F3, F4) from hawthorn seeds on ROS generation in human colonocytes (CCD841 CoN) treated with H_2_O_2_. Ascorbic acid (AA) at a concentration of 10.0 μg/mL. Statistically significant differences compared to the control (H_2_O_2_ treated cells) are marked with * and ** for *p* < 0.05, *p* < 0.01, respectively.

Furthermore, the improved colonocyte protection provided by F2 was also confirmed in the ROS generation assay. ROS are chemically active oxygen-containing molecules produced in cells during cellular respiration, among other processes. In excess, they can damage proteins, lipids, and DNA [2]. For these reasons, measurement of ROS generation by cells was performed to assess the ability of the seed preparations to intervene against oxidative damage induced by H_2_O_2_. In this test, the lowest intracellular ROS generation by colonocytes, i.e., the highest antioxidant activity, was demonstrated for F2 (Figure 4). Treatment of colonocytes with H_2_O_2_ increased ROS levels to 178% compared to the control (100%). However, simultaneous treatment of cells with H_2_O_2_ and F2 at the highest dose tested reduced ROS generation to 111%. These results indicate that F2 has the ability to mitigate ROS generation and reduce the occurrence of oxidative damage. These properties may be attributed to its dominant proanthocyanidins. Previously, this group of compounds has been shown to enhance the antioxidant protection of cells via the MAPK pathway, regulating the expression of genes and proteins involved in the NF-κB pathway [33], leading to an increase in the expression of antioxidant enzymes [34] and stabilization of mitochondria [35], inducing the expression of Nrf2, which activates HO-1 (hemoxygenase-1) and NQO1 (NAD(P)H quinone dehydrogenase 1), and reducing the expression of apoptosis-related genes (the ratio of Bax and Bcl2) to protect cells [36]. The chemopreventive properties of proanthocyanidins make F2 an effective free radical scavenger. F2 and F3 have been shown to have lower cellular antioxidant activity despite also containing proanthocyanidins. A similar relationship was previously reported by Defour et al. [13], who fractionated a chokeberry fruit extract. This study demonstrated the highest ROS inactivation capacity in a cellular model for the proanthocyanidin fraction, while combining proanthocyanidins and phenolic acids in a different fraction resulted in their antagonistic effects, leading to reduced antioxidant efficacy.

Oxidative stress and chronic inflammation are the causes of many chronic diseases of the large intestine, such as inflammatory bowel disease (Crohn’s disease and ulcerative colitis) and colon cancer [1]. Therefore, in the next stage of our research, we analyzed the effect of polyphenolic fractions from hawthorn seeds on LPS-induced NO generation by macrophages (RAW 264.7 cell line). The in vitro model with RAW 264.7 cells is the most frequently used research system in the screening of anti-inflammatory compounds due to the strong production of inflammatory biomarkers under the influence of inflammatory stimulators [37].

**Figure 5:**
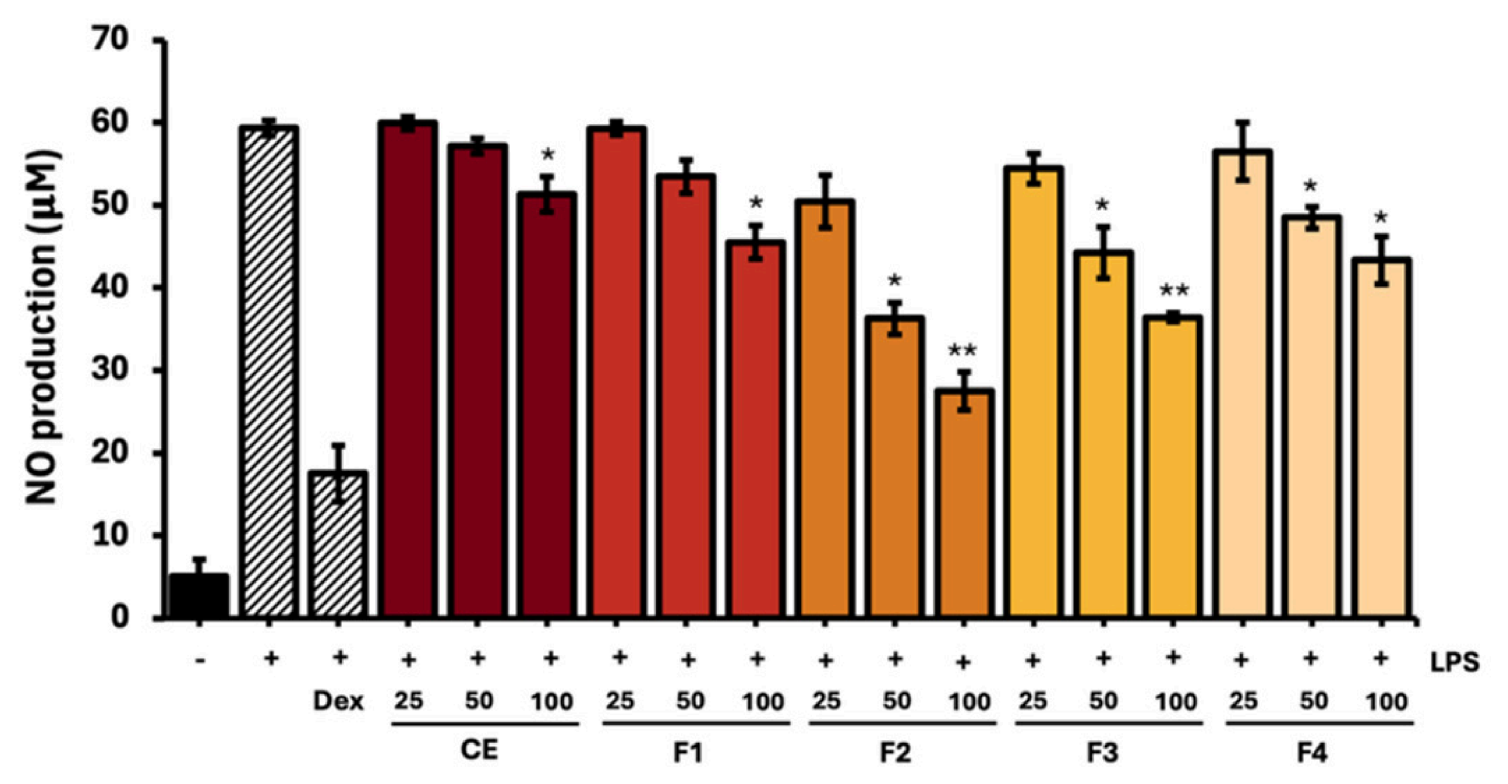
Effect of crude extract (CE) and polyphenolic fractions (F1, F2, F3, F4) from hawthorn seeds on NO generation by murine macrophages (RAW264.1). Dexamethasone (Dex) at a concentration of 20 μM. Statistically significant differences compared to the control (H_2_O_2_ treated cells) are marked with * and ** for *p* < 0.05, *p* < 0.01, respectively.

In our anti-inflammatory activity studies, the highest activity was again demonstrated for F2. Compared to the control, treatment of RAW 264.7 cells with F2 resulted in a dose-dependent decrease in LPS-induced NO production by 27, 43, and 68% for concentrations of 25, 50, and 100 µg/mL, respectively (Figure 5). Remarkably, at the highest administered dose, this result was only 10 µM higher compared to the positive control (Dexomethasone, 20 µM). The anti-inflammatory activity of the remaining preparations administered at a dose of 100 µg/mL can be ranked in the order F3 > F4 > F1 > CE. Previously, proanthocyanidins isolated from grape seeds administered at a dose of 80 µg/mL were shown to reduce NO by 29.2% in RAW 264.7 cells [38]. In vivo studies in mice confirmed the anti-inflammatory properties of proanthocyanidins, which resulted from the reduction in ROS production, suppression of the NLRP3 infamasome and polarization of M1 macrophages [39,40].

The above results suggest that among all the polyphenolic fractions of hawthorn seeds, F2 may help shape the redox environment, contributing to the protection of the colon epithelium against oxidative stress, excessive ROS production and the development of an inflammatory response.

### 2.4. Cytotoxic, Antimigratory and Antiinvasive Activity of the Fraction Against Cancer Cells

Four human colon cancer cell lines differing in their differentiation rate, proliferation rate, response to treatment, and metastasis potential were selected for cytotoxic activity analysis. We believe this approach allows for better identification of therapy efficacy within the broader context of tumor heterogeneity. We selected Caco-2, Ht-29, Dld-1, and Ls180 cell lines for analysis. Cisplatin was used as a positive control (Table 2).

As can be seen in Table 2, regardless of the treated cell line, F2 demonstrated the highest cytotoxic activity. The IC_50_ ranged from 39.9 (Ht-29) to 100.7 (Dld-1) µg/mL. The potency of the remaining preparations can be ranked in the following order: F3 > F4 > CE > F1. Interestingly, fractionation did not increase the cytotoxic activity of F1. This can be explained by the predominant concentration of phenolic acids in this preparation. Numerous previous reports have confirmed the low cytotoxic efficacy of phenolic acids against colon cancer cell lines SW620 [12], Caco-2 [41], SW-480 and HT-29 [42]. Also previously, Navarro et al. [12] fractionated *Uncaria tomentosa* leaves on Diaion HP-20 resin, obtaining five fractions differing in polyphenolic composition. In their cytotoxicity studies against colon cancer cells, the authors also demonstrated activity dependent on the content of procyanidins oligomers. For the fraction rich in these compounds (25.0 mg/g of extract), the IC_50_ value was 71.4 µg/mL, which was 2.9 times lower compared to the fraction rich in phenolic acids (2.8 mg/g of extract). Also, for the proanthocyanidin extract from hawthorn fruit, the IC_50_ value after 24 and 48 h was 321.6 and 300.0 µg/mL, respectively, against colon cancer cells (HCT116) [20]. Preparation F2 also demonstrated preferential cytotoxicity towards colon cancer cells, as indicated by selectivity index (SI) values ranging from 1.0 to 2.5 obtained against normal colonocytes and CCD841CoN cancer cells (Table S4).

In summary, the sensitivity of the cell lines to the tested preparations depended on the type of fraction, indicating a high selectivity of the preparations among the tested cell lines. Therefore, it can be assumed that this selectivity was strongly influenced by the polyphenolic composition of the preparations.

**Figure 6:**
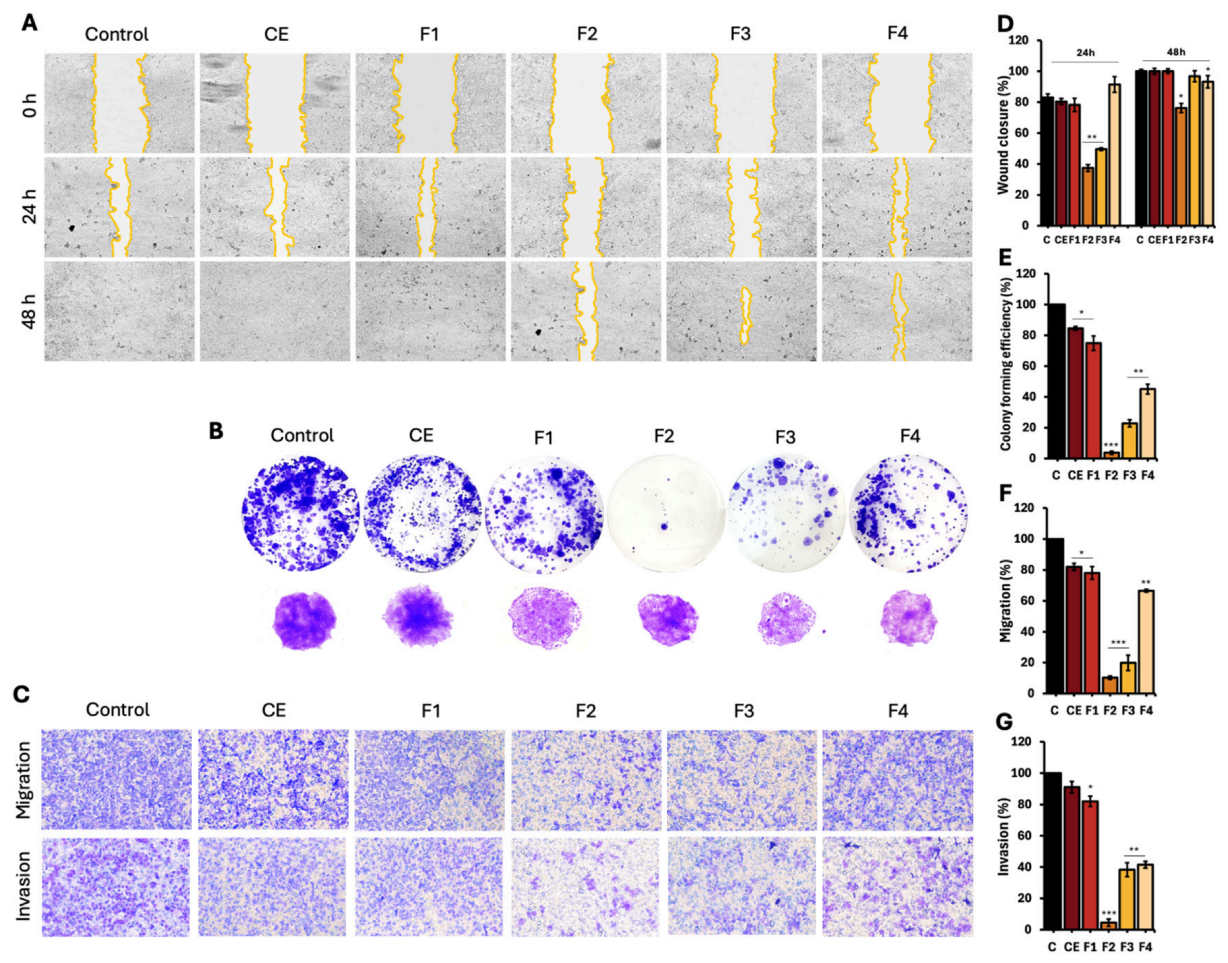
Activity of crude extract (CE) and polyphenolic fractions (F1, F2, F3, F4) in the wound (**A**,**D**), colony (**B**,**E**), migration (**C**,**F**) and invasion (**C**,**G**) assays. Statistically significant differences compared to the control (H_2_O_2_ treated cells) are marked with *, ** and *** for *p* < 0.05, *p* < 0.01 and *p* < 0.001, respectively.

Among the selected colon cancer cell lines, Dld-1 has the highest metastatic potential and was therefore selected for further analysis. Using this line, polyphenolic seed preparations were next analyzed for their ability to inhibit cancer cell proliferation, migration, and invasion. This potential was assessed using the wound assay, clonogenic assay, migration assay, and Transwell chamber invasion assay. The preparations were tested at the IC_25_ (75% cell viability) dose (Table S3) in the wound assay and the IC_50_ dose in the remaining assays (Figure 6).

One of the hallmarks of cancer cells is their potential for unlimited multiplication and self-renewal. A clonogenic assay is a test that assesses the effectiveness of a study drug in killing cancer cells and inhibiting their ability to grow further [43]. In this test, the highest efficacy in inhibiting Dld-1 cell growth was demonstrated for F2, where the colony formation value was 3.7% compared to the control (100%). The activity of the remaining preparations can be ranked in the order F3 > F4 > F1 > CE, with no statistically significant difference compared to the control demonstrated for CE and F1. This once again confirms the low therapeutic efficacy of seed phenolic acids, coupled with the high proanthocyanidin content.

In a further phase of this study, the potential of the preparations to inhibit cancer cell migration and invasion was assessed. Within a cancer tumor, cells can migrate or invade surrounding tissues, and then spread to distant sites in the body, creating new tumor foci. Cancer cell metastasis is the cause of death for nearly 67% of cancer patients [44]. Therefore, the search for agents that interfere with this ability of cancer cells is an essential part of cancer research. In our studies, F2 showed the highest potential for inhibiting Dld-1 cell migration and invasion. In the wound assay, F2 demonstrated the ability to inhibit cell migration by 37.4% and 76.2% after 24 and 48 h, respectively, compared to the control. These results were confirmed in the Transwell migration assay, where migration was inhibited by 10.2%. In the invasion assay, F2 inhibited cell invasion by 4.5%. In previous studies, Wang et al. [20] showed that the level of cell migration and invasion through the Transwell chamber was 71.5% and 82.6%, respectively, for proanthocyanidin extract from hawthorn fruit at a dose of 200 µg/mL. In our studies, significantly lower doses demonstrated significantly better results. Furthermore, better results were obtained compared to grape seed proanthocyanidin extract, where a concentration of 50 µg/mL demonstrated the ability to inhibit the migration and invasion of transwell bladder cancer cells (T24 cell line) by 67.5% and 45.1%, respectively [19]. The above anticancer properties of proanthocyanidins are mediated through various signaling pathways. It has been shown that they can reduce the expression of kinases involved in the PI3K/Akt/mTOR (phosphoinositide 3-kinase/protein kinase B/mammalian target of rapamycin) pathway, leading to the induction of apoptosis and autophagy [45,46]. They also activate the mitochondrial apoptosis pathway by increasing the Bax/Bcl-2 ratio, losing mitochondrial membrane potential and increasing the activity of casase-3 and -9 [47]. Additionally, they can modulate MAPK (JNK, p38) and NF-κB pathways and influence the cell cycle, arresting cells in the G1 or G2/M phase [48,49]. In turn, the inhibition of cancer cell metastasis by PCA has been attributed to the inhibition of EMT (epithelial-to-mesenchymal transition) associated with MMP-9 (matrix metalloproteinase-9) and inactivation of the Wnt/β-catenin signaling pathway [20,36]. The above data explain the cytotoxic effect of hawthorn seed fractions on colon cancer cells and emphasize the potential of the obtained fractions in comparison to other fruit seed extracts analyzed so far.

However, although fraction F2 demonstrated significant biological activity in vitro, the concentrations used in this study should not be directly extrapolated to physiological conditions. Flavan-3-ols, and especially high-molecular-weight proanthocyanidins, are characterized by limited absorption in the upper gastrointestinal tract, allowing a significant portion to reach the colon, where they undergo extensive microbial biotransformation to low-molecular-weight phenolic metabolites. These metabolites may also contribute to the overall biological activity observed in vivo. Therefore, further studies involving simulated gastrointestinal digestion, gut microbiome metabolism, and in vivo models are necessary to confirm the physiological relevance of these findings [50,51].

### 2.5. PCA

**Figure 7:**
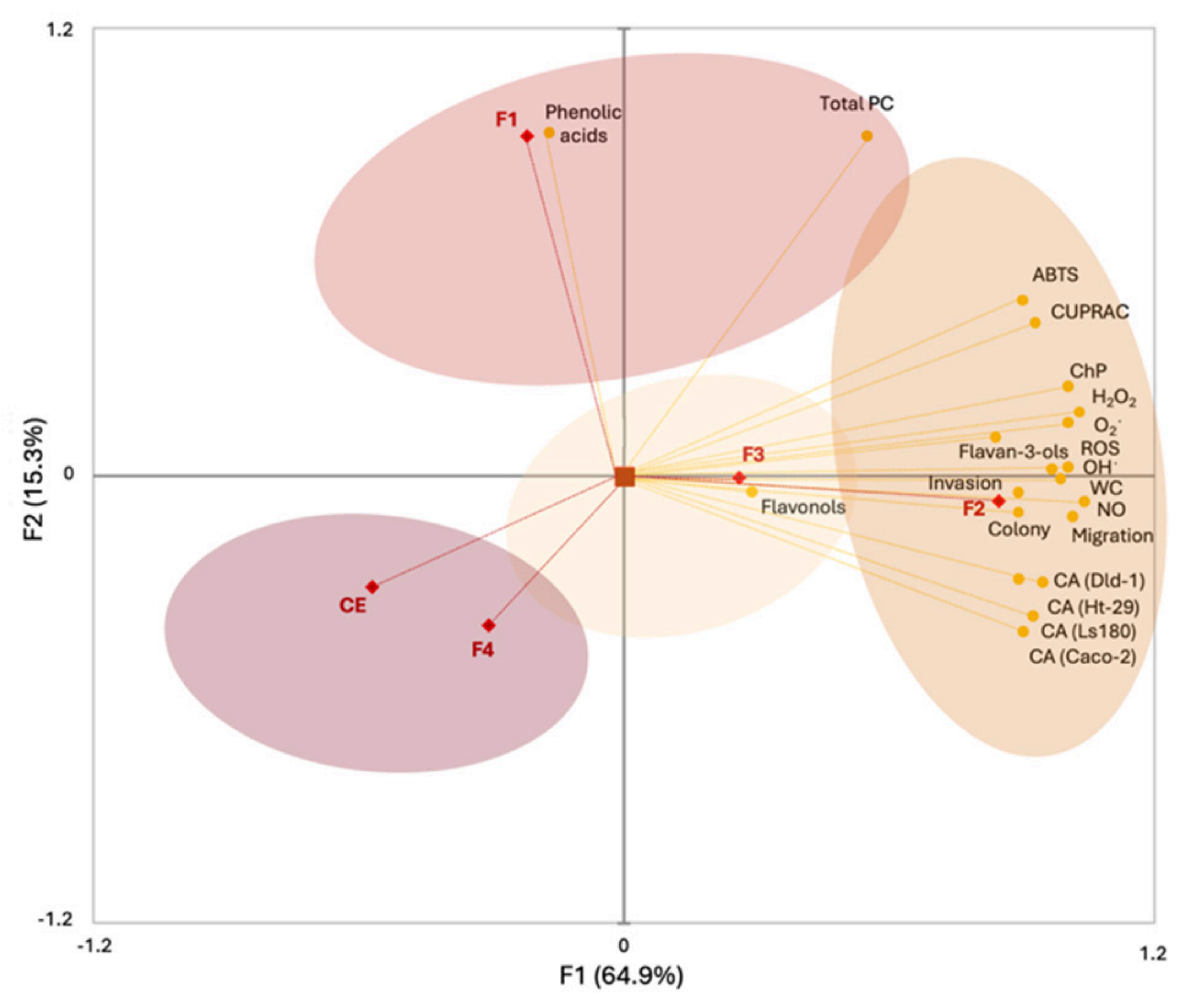
PCA analysis between the tested preparations and the analyzed parameters.

Principal component analysis (PCA) is a statistical analysis that allows for the elucidation of the relationships between the studied samples and the analyzed parameters. In our study, principal components explained 64.9% and 15.3% of the total data variability, respectively (Figure 7). The PCA analysis identified four groups with similar phytochemical and biological properties. The first group was represented by formulation F1 and its dominant phenolic acids. The second group was formed by formulations CE and F4, which exhibited a similar profile of the studied parameters. The third group included formulation F3, in which flavonoids were the dominant class of compounds. The final, fourth group included the F2 preparation and the remaining analyzed parameters, i.e., antioxidant activity in the chemical model and cellular model, ROS and NO scavenging, cytotoxic activity, anti-colony formation, anti-migration, and anti-invasive activity. These parameters correlated with the high content of flavan-3-ols. Based on the PCA analysis, it can be concluded that the degree of fractionation of the raw seed extract significantly affects the quantitative and qualitative composition of polyphenolic compounds and the biological activity of the obtained preparations. Phenolic acids contributed most to PC2, while flavan-3-ols, antioxidant activity, and anticancer properties were the main contributors to PC1. Furthermore, F2, characterized by the most favorable values of the analyzed parameters, could, after further in vitro and in vivo studies, have broad applications in the nutraceutical and pharmaceutical industries.

## 3. Materials and Methods

### 3.1. Plant Material

The study material consisted of hawthorn seeds of the *C. monogyna* species purchased at retail. The seeds were manually separated and ground into a powder.

### 3.2. Extraction and Fractionation

The crushed seeds (1.5 kg) were extracted with three times increasing concentrations of methanol (50, 75, and 96%), each time assisted by ultrasonication (Sonic 10 bath, Polsonic, Poland) for 30 min at 30 °C and 40 kHz [7,8]. After each step, the extracts were centrifuged (type 5430, Eppendorf, Hamburg, Germany), and the combined supernatants were evaporated using a rotary evaporator (R-215 Rotavapor, Buchi, Switzerland). A portion of the pre-evaporated extract was lyophilized to powder, obtaining the crude extract (CE), while the remaining portion was used for fractionation. Polyphenolic fractions from CE were extracted using a glass column (3.5 × 10 cm) packed with LiChroprep C-18 resin (40–63 µm). After conditioning, CE was applied to the bed. Compounds other than polyphenols were eluted with water, and then fractionation was performed using 20% methanol (F1), 60% (F2), 80% (F3), and 96% (F4). The resulting fractions, after evaporation, were lyophilized into powder.

For the analysis of antioxidant activity in the chemical model and UPLC analysis, CE, F1, F2, F3, and F4 preparations were dissolved in 50% methanol and passed through a 0.45 μm filter. For the cellular analyses, the preparations were dissolved in 30% DMSO and passed through a 0.20 μm filter.

### 3.3. Polyphenol Profile Analysis

Determination of polyphenolic compounds was carried out using the Ultra-Performance Liquid Chromatography (UPLC) Waters ACQUITY system (Waters, Milford, MA, USA). UPLC was equipped with a binary pump manager, column manager, sample manager, photodiode array (PDA) detector, tandem quadrupole mass spectrometer (TQD) with electrospray ionization (ESI) source. Separation of polyphenols was performed using a 1.7 µm, 100 mm × 2.1 mm UPLC BEH RP C18 column (Waters, Milford, MA, USA). The mobile phase consisted of water (solvent A) and 40% acetonitrile (solvent B). The flow rate was kept constant at 0.35 mL/min for a total run time of 8 min. The system was run with the following gradient program: from 0 min 5% B, from 0 to 8 min linear to 100% B, and from 8 to 9.5 min for washing and back to initial conditions. The injection volume of the samples was 5 µL, and the column was supported at 50 °C. The following TQD parameters were used: cone voltage of 30 V, capillary voltage of 3500 V, source and desolvation temperature 120 °C and 350 °C, respectively, and desolvation gas flow rate of 800 L/h. Characterization of the individual polyphenolic compounds was performed on the basis of the retention time, mass-to-charge ratio, fragment ions, and comparison of data obtained with commercial standards and literature findings. Obtained data were processed in Waters MassLynx v.4.1 software (Waters, Milford, MA, USA) [8]. The results are expressed in mg/g dm.

### 3.4. Antioxidant Activity Analysis

Five tests were selected to evaluate the antioxidant activity in the chemical model of the preparations: ABTS^•^+ radical scavenging activity (ABTS), copper ion reduction (CUPRAC), superoxide radical scavenging activity (O_2_^•−^), hydroxyl radical scavenging activity (OH^•^), and measurement of metal ion chelating potential (ChP).

The scavenging activity of extract on ABTS (2,2′-azino-bis(3-ethylbenzothiazoline-6-sulfonic acid) radicals was assessed according to the method of Re et al. [52]. The extract was mixed with ABTS solution and left for 6 min. The absorbance was measured at the 734 nm.

The CUPRAC test was assessed according to the method described by Apak et al. [53]. The extract was mixed with neocuproine (7.5 mM), acetate buffer (1 M, pH 7.0), copper chloride (10 mM), and left for 30 min. The absorbance was measured at the 450 nm.

The O_2_^•−^ radical scavenging activity was measured using the method described by Robak and Gryglewski [54]. The extract was mixed with NBT (150 µM), NADH (468 µM), PMS (60 µM), and left for 5 min. The absorbance was measured at the 560 nm.

The OH^•^ radical scavenging activity was based on the method of Halliwell et al. [55]. The extract was mixed with 2-deoxyribose (0.2 mM), EDTA (1.04 mM), iron ammonium sulphate (1.0 mM), perhydrol (0.1 M), and ascorbic acid (1.0 mM). The solution was left for 1 h, then thiobarbituric acid (1%, *w*/*v*) and trichloroacetic acid (2.8%, *w*/*v*) were added and it was left for 15 min. The absorbance was measured at the 532 nm.

The ChP was determined by the method described by Mosmann [56]. The extract was mixed with ferrozine (0.25 mM), iron sulfate (0.1 mM) and left for 10 min. The absorbance was measured at the 562 nm.

The results are expressed as mmol Trolox (TE)/g d.m and as IC_50_ (μg/mL). Positive controls included quercetin, EDTA and ascorbic acid.

### 3.5. Cell Viability Assessment

Cell viability assessment was performed according to our previous reports [57]. Trypsinized cell lines were seeded into 96-well plates at a density of 8 × 10^3^ cells per well and placed in an incubator for 24 h. After cell adhesion, the culture medium was removed, and the test preparations diluted in culture medium were added for 24 h. After this time, the MTS assay was performed according to the manufacturer’s instructions (Promega, Madison, WI, USA). The results are expressed as IC_50_ (μg/mL). Cisplatin was used as a positive control. Based on the obtained IC_50_ values, the selectivity index (SI) was also calculated according to the formula SI = IC_50_ (healthy cells)/IC_50_ (cancer cells).

### 3.6. Assessment of Antioxidant Activity in the Cellular System

This study was performed on CCD841 CoN cell line, in accordance with our previous report [57]. Caco-2 (ATCC® HTB-37), Ht-29 (ATCC® HTB-38), Dld-1 (ATCC® CCL-221), Ls180 (ATCC® CL-187), CCD 841 CoN (ATCC® CRL-1790) and RAW 264.7 (ATCC® TIB-71) cell lines were obtained from the American Type Culture Collection (ATCC, Rockville, MD, USA). Briefly, in the first stage, cell sensitivity to H_2_O_2_ (0.008–0.5 mM) was determined. Cell viability was assessed according to the procedure described in Section 3.5. Based on the obtained results, the appropriate H_2_O_2_ concentration was selected for further experiments. In the second stage, cells were seeded into 96-well plates at a concentration of (1 × 10^4^ cells/well) for 24 h. After this time, the cells were incubated with the tested preparations for 12 h and then treated with H_2_O_2_ at a concentration of 0.125 mM. After incubation, cell viability was assayed according to Section 2.5. The data were presented as % cell viability. Ascorbic acid served as a positive control.

### 3.7. Reactive Oxygen Species (ROS) Generation Test

The determination of ROS levels in CCD841 CoN cells was performed based on the method described by Sanches et al. [58]. Cells were seeded into 12-well plates at a density of 1 × 10^5^ cells per well for 24 h. Cells were then exposed to the combination of the tested preparations with 0.125 mM H_2_O_2_. After incubation, the fluorescent dye 2′,7′-dichlorofluorescein diacetate (DCFH-DA) was added at a concentration of 10 μM. After 0.5 h of incubation, fluorescence intensity was recorded at an excitation wavelength of 484 nm and an emission wavelength of 535 nm. The obtained results are expressed as the % of reactive oxygen species produced. Ascorbic acid was used as a positive control.

### 3.8. Nitric Oxide (NO) Generation Measurement Test

The determination of ROS levels in RAW 264.7 cells was performed based on the method described by Grzelczyk et al. [59]. Cells were seeded into 96-well plates at a density of 1 × 10^5^ cells per well for 24 h. Cells were then stimulated with lipopolysaccharide (LPS, O55:B5 from *E. coli*) at a concentration of 1 μg/mL for another 24 h. After this period, the tested preparations were added to the cultures for 12 h. After this time, Griess reagent was added to the collected supernatant, and absorbance at 550 nm was recorded after 15 min. The obtained results are expressed as sodium nitrite concentration. Dexamethasone was used as a positive control.

### 3.9. Wound Test

This study was performed on the Dld-1 cell line, consistent with our previous report [57]. Cells were seeded into 12-well plates at a density of 2 × 10^4^ cells per well and cultured until approximately 90% confluence was achieved. Linear disruption of the cell monolayer was then performed using a sterile pipette tip. The test preparations were added to the cultures at IC_25_ (75% cell viability) concentrations. Cell migration was observed at 0, 24, and 48 h after wound creation, and images were captured using an inverted light microscope (Oxion Inverso, Euromex, Mataro, Spain). The results are expressed as a percentage of the wound closure area relative to the control (untreated cells).

### 3.10. Colony-Forming Ability Test

This study was performed on the Dld-1 cell line, consistent with our previous report [57]. Cells were seeded into 6-well plates at a density of 5 × 10^2^ cells per well for 24 h. Cells were then exposed to the test preparations diluted in medium for 12 h. After incubation, the medium containing the preparations was removed, and cultures were continued for the next 14 days, with regular renewal. The resulting cell colonies were fixed with chilled ethanol, stained with 0.5% crystal violet, and then counted. The results are expressed as a percentage of the number of colonies relative to the control (untreated cells).

### 3.11. Cell Migration and Invasion Assay

This study was performed on the Dld-1 cell line, consistent with our previous report [57]. Cells were seeded onto inserts (8 μm pore diameter) at a density of 2.4 × 10^4^ cells per well placed in 24-well plates. For analysis of cell invasion, the surface of the inserts was previously covered with a layer of Martigel. The tested preparations were applied to both the upper and lower portions of the inserts. The prepared systems were then incubated for 48 h. Migrated and invaded cells were fixed in 4% paraformaldehyde and stained with 0.5% crystal violet. The obtained results are presented as a percentage of migratory and invasive cells in relation to the control sample (untreated cells).

### 3.12. Statistical Analysis

The obtained results are presented as mean values with their corresponding standard deviations (SD). Statistical analyses were performed using Statistica version 13.3 (StatSoft, Krakow, Poland). One-way analysis of variance (ANOVA) with Tukey’s post hoc test and Duncan’s test was used. Additionally, principal component analysis (PCA) was performed.

## 4. Conclusions

The novelty of this study was the demonstration that hawthorn seeds, a waste and underutilized material, can be an interesting plant matrix for obtaining polyphenolic preparations with targeted activity against healthy and cancerous colon cells. Fractionation on C18 resin using four solvent concentrations allowed for the production of preparations with concentrated content of specific polyphenolic compounds in a small mass. Phenolic acids dominated in F1, flavan-3-ols in F2, and flavonoids in F3 and F4. This selective polyphenol distribution had a significant impact on the estimated health-promoting activity. In the assessment of antioxidant activity in a chemical model, the highest potential was demonstrated for F2, which was also confirmed in a cellular model using human colonocytes. F2 also demonstrated the highest cytotoxic potential against colon cancer cells, inhibiting their proliferation, migration, and invasion.

The limitations of this work include the in vitro model and the lack of an estimate of the activity of the fraction truly bioavailable after digestion. Therefore, future research should address these shortcomings, which would allow for a full understanding of the bioactive potential of hawthorn seeds. Another interesting solution would be to suspend the most active fraction in a specially developed oral formulation, allowing for the protection of polyphenolic compounds in the upper gastrointestinal tract while targeting their activity in the large intestine. Such a solution could have broad nutraceutical applications in the prevention and treatment of colon diseases.

## Figures and Tables

**Table 1:** Content of polyphenolic compounds in the crude extract (CE) and polyphenolic fractions (F1, F2, F3, F4) from hawthorn seeds.

No	Compound	Amount (mg/g dw)				
		CE	F1	F2	F3	F4
1 *	Gallic acid	-	7.5 ± 0.2	-	-	-
2	Ellagic acid derivative	-	8.7 ± 0.1	-	-	-
3	Coumaric acid derivative	-	16.3 ± 0.2	-	-	-
4	Vanillic acid glucoside	0.6 ± 0.0	19.0 ± 0.2	-	-	-
5	5-*O*-caffeoylquinic acid	1.9 ± 0.0	30.1 ± 0.3	-	-	-
6	(Epi)afzelechin-(Epi)catechin isomer I	-	5.9 ± 0.1	-	-	-
7	(Epi)afzelechin-(Epi)catechin isomer II	-	2.1 ± 0.1	-	-	-
8	Coumarylquinic acid isomer I	0.1 ± 0.0	38.9 ± 0.3	-	-	-
9	3-*O*-caffeoylquinic acid	1.5 ± 0.1	9.2 ± 0.1	-	-	-
10	4-*O*-caffeoylquinic acid	0.6 ± 0.0	5.3 ± 0.1	-	-	-
11	Procyanidin dimer type-B	-	8.1 ± 0.1	-	-	-
12	Procyanidin dimer type-B	-	1.8 ± 0.1	-	-	-
13	Vanillic acid derivative	-	2.0 ± 0.1	-	-	-
14	(+)catechin	3.2 ± 0.1	-	28.3 ± 0.4	-	-
15	Procyanidin trimer type-C	-	-	4.8 ± 0.1	-	-
16	Coumarylquinic acid isomer II	3.1 ± 0.0	-	12.1 ± 0.2	-	-
17	Procyanidin tetramer	2.3 ± 0.0	-	23.6 ± 0.3	-	-
18	Procyanidin trimer type-C	0.9 ± 0.0	-	9.9 ± 0.2	-	-
19	Proanthocyanidin dimer glucoside I	0.5 ± 0.0	-	5.7 ± 0.1	-	-
20	Procyanidin trimer type-C	0.3 ± 0.0	-	4.6 ± 0.1	-	-
21	Vanillic acid derivative	0.2 ± 0.0	-	2.2 ± 0.1	1.6 ± 0.1	-
22	Kaempferol-*O*-galloyl-pentoside isomer I	0.8 ± 0.0	-	1.1 ± 0.0	3.9 ± 0.2	0.2 ± 0.0
23	Unspecified (Epi)afzelechin derivative	0.2 ± 0.0	-	5.7 ± 0.2	-	-
24	Proanthocyanidin dimer glucoside I	0.4 ± 0.0	-	8.7 ± 0.3	-	-
25	Kaempferol-*O*-galloyl-pentoside isomer II	0.4 ± 0.0	-	0.7 ± 0.0	7.0 ± 0.2	-
26	Kaempferol-*O*-galloyl-pentoside isomer III	0.2 ± 0.0	-	2.99 ± 0.05	7.1 ± 0.0	0.5 ± 0.1
27	Quercetin *O*-acetyl-hexoside	0.2 ± 0.0	-	1.0 ± 0.0	6.0 ± 0.1	1.0 ± 0.0
28	Unspecified Quercetin derivative	-	-	0.5 ± 0.0	55.1 ± 0.1	2.0 ± 0.0
	Sum of phenolic acids	8.9 ± 0.1 ^b^	136.9 ± 1.0 ^d^	14.3 ± 0.6 ^c^	1.6 ± 0.3 ^a^	-
	Sum of flavan-3-ols	8.0 ± 0.1 ^a^	18.0 ± 0.5 ^b^	91.3 ± 2.1 ^c^	-	-
	Sum of flavonols	1.8 ± 0.1 ^a^	-	6.3 ± 0.2 ^c^	79.3 ± 1.2 ^d^	3.7 ± 0.2 ^b^
	TOTAL (mg/g)	18.7 ± 0.2 ^b^	154.9 ± 1.5 ^e^	111.9 ± 2.9 ^d^	80.9 ± 1.5 ^c^	3.7 ± 0.2 ^a^

Explanations: *, identification of compounds according to Table S1; -, not identified. Determinations were performed in triplicate. Statistically significant differences between samples are marked with different letters (a–e) for *p* < 0.05.

**Table 2:** IC_50_ values (µg/mL) of crude extract (CE), polyphenolic fractions (F1, F2, F3, F4) from hawthorn seeds and positive control (Cisplatine) against colon cancer cell lines.

Cell Line	CE	F1	F2	F3	F4	Cisplatine
			IC_50_ (μg/mL)			
Caco-2	390.7 ± 8.1 ^f^	301.8 ± 6.3 ^e^	47.2 ± 1.0 ^b^	143.0 ± 2.1 ^d^	126.3 ± 2.6 ^c^	1.9 ± 0.2 ^a^
Ht-29	408.2 ± 8.5 ^e^	721.5 ± 14.9 ^f^	39.9 ± 0.8 ^b^	62.5 ± 0.9 ^c^	152.5 ± 3.2 ^d^	12.8 ± 0.4 ^a^
Dld-1	470.2 ± 9.7 ^e^	551.4 ± 5.6 ^f^	100.7 ± 2.1 ^b^	119.3 ± 1.7 ^c^	158.1 ± 3.3 ^d^	3.3 ± 0.2 ^a^
Ls180	359.9 ± 7.5 ^d^	630.6 ± 13.1 ^e^	40.0 ± 1.1 ^b^	90.3 ± 1.3 ^c^	91.4 ± 1.9 ^c^	8.4 ± 0.3 ^a^

Statistically significant differences between samples are marked with different letters (a–f) for *p* < 0.05.

## Data Availability

Data are available from the authors.

## Supplementary Materials

The following supporting information can be downloaded at: https://www.mdpi.com/article/10.3390/molecules31152694/s1, Table S1: Polyphenolic composition of crude extract (CE) and polyphenolic fractions (F1,F2, F3 and F4) from hawthorn seeds; Table S2: Positive control values for tests assessing antioxidant activity; Figure S1: Effect of H_2_O_2_ on CCD841CoN cell viability. Cells were treated with different concentrations of H_2_O_2_ (0.008 to 2.5 mM); Table S3: IC25 (75% cell viability) values (µg/mL) of crude extract (CE) and polyphenolic fractions (F1, F2, F3, F4) from hawthorn seeds against colon cancer cell lines. Table S4. Selectivity index (SI) between IC_50_ values for healthy cells (CCD841 CoN line) and cancer cells.
