# Supplementary material for: Fractionation-Based Modeling of Hawthorn (Crataegus L.) Preparation Bioactivity: Relationship Between Fraction Composition and Mechanisms of Action in Colon Cells
Source: Molecules. 2026 Aug 3;31(15):2694. doi: 10.3390/molecules31152694 (PMC13468617; doi:10.3390/molecules31152694)
Supplement: Supplementary file 1 [file molecules-31-02694-s001.zip › molecules-4382924-supplementary.pdf]

Supplementary material

Fractionation-based modeling of hawthorn (*Crataegus* L.) preparation bioactivity: relationship between fraction composition and mechanisms of action in colon cells

Natalia Żurek*, Ireneusz Kapusta

Department Food Technology and Human Nutrition, Institute of Food Technology and Nutrition, University of Rzeszow, 4 Zelwerowicza St., 35-601 Rzeszow, Poland;

* Correspondence: nzurek@ur.edu.pl; Tel.: +48-17-785-5236

Content

Table S1. Polyphenolic composition of crude extract (CE) and polyphenolic fractions (F1,F2, F3 and F4) from hawthorn seeds.
Table S2. Positive control values for tests assessing antioxidant activity.
Figure S1. Effect of H_2O_2 on CCD841CoN cell viability. Cells were treated with different concentrations of H_2O_2 (0.008 to 2.5 mM).
Table S3. IC_{25} (75% cell viability) values (µg/mL) of crude extract (CE) and polyphenolic fractions (F1, F2, F3, F4) from hawthorn seeds against colon cancer cell lines.
Table S4. Selectivity index (SI) between IC_{50} values for healthy cells (CCD841 CoN line) and cancer cells.

Table S1. Polyphenolic composition of crude extract (CE) and polyphenolic fractions (F1,F2, F3 and F4) from hawthorn seeds.

No	Compound	Rt	λ_{max}	[M-H]⁻ m/z	
		min	nm	MS	MS/MS
1	Gallic acid	1.55	277	169	125
2	Ellagic acid derivative	1.71	281	347	301
3	Coumaric acid derivative	1.88	277	361	163
4	Vanillic acid glucoside	2.14	252, 290	329	167
5	5-*O*-caffeoylquinic acid	2.40	324	353	191
6	(Epi)afzelechin-(Epi)catechin isomer I	2.44	279	561	409
7	(Epi)afzelechin-(Epi)catechin isomer II	2.59	279	561	409
8	Coumarylquinic acid isomer I	2.77	310	337	191
9	3-*O*-caffeoylquinic acid	2.82	324	353	191
10	4-*O*-caffeoylquinic acid	2.88	324	353	191
11	Procyanidin dimer type-B	3.29	278	577	425, 289
12	Procyanidin dimer type-B	3.31	278	577	425, 289
13	Vanillic acid derivative	3.45	260, 290	421	197
14	(+)Catechin	3.98	278	289	-
15	Procyanidin trimer type-C	4.21	278	865	577
16	Coumarylquinic acid isomer II	4.31	312	337	163
17	Procyanidin tetramer	4.37	278	1155	865
18	Procyanidin trimer type-C	4.44	278	865	577
19	Proanthocyanidin dimer glucoside I	4.58	279	721	559, 433
20	Procyanidin trimer type-C	4.78	278	865	577
21	Vanillic acid derivative	5.25	262, 290	373	167
22	Kaempferol-*O*-galloyl-pentoside isomer I	5.41	285, 337	569	417
23	Unspecified (Epi)afzelechin derivative	5.85	276	551	405, 273
24	Proanthocyanidin dimer glucoside I	5.94	279	721	559, 433
25	Kaempferol-*O*-galloyl-pentoside isomer II	6.63	285, 337	569	417
26	Kaempferol-*O*-galloyl-pentoside isomer III	6.69	285, 337	569	417
27	Quercetin *O*-acetyl-hexoside	7.86	281, 345	505	463, 301
28	Unspecified Quercetin derivative	9.10	345	337	301

Table S2. Positive control values for tests assessing antioxidant activity.

Test	Ascorbic acid (mmol TE/g)	Quercetin (mmol TE/g)	EDTA (IC_{50}, μg/mL)
ABTS	4.1 ± 0.0	-	-
CUPRAC	70.2 ± 0.3	-	-
$OH^{\bullet}$	-	11.1 ± 0.0	-
$O_2^{\bullet-}$	83.0 ± 1.0	-	-
ChP			14.4 ± 0.5

Values are presented as means and SD (n=3). -, not marked.

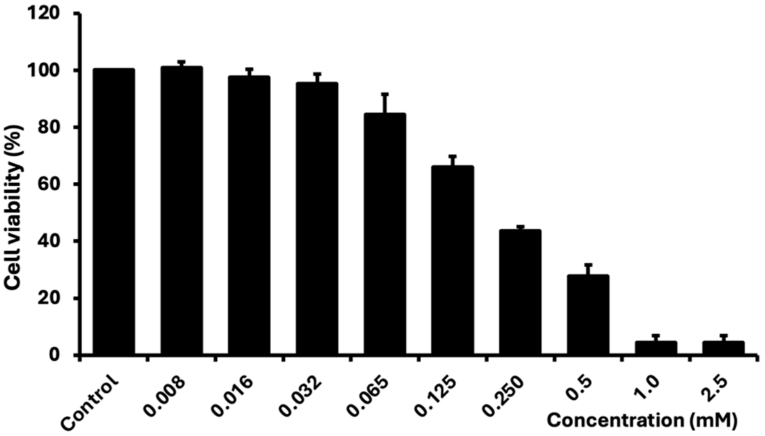

Figure S1. Effect of H_2O_2 on CCD841CoN cell viability. Cells were treated with different concentrations of H_2O_2 (0.008 to 2.5 mM). Cell viability was assessed by MTS assay. Values are presented as means and SD (n=9).

Table S3. IC_{25} (75% cell viability) values (μg/mL) of crude extract (CE) and polyphenolic fractions (F1, F2, F3, F4) from hawthorn seeds against colon cancer cell lines.

No	Cell line	CE	F1	F2	F3	F4
				IC_{25} (μg/mL)		
1	Caco-2	210.6 ± 1.8	106.5 ± 0.9	6.6 ± 0.9	52.6 ± 1.2	35.1 ± 1.7
2	Ht-29	302.7 ± 3.2	526.8 ± 2.8	7.1 ± 0.8	8.4 ± 0.4	84.3 ± 0.3
3	Dld-1	369.4 ± 4.4	372.5 ± 3.1	68.5 ± 1.1	58.7 ± 1.5	84.0 ± 0.7
4	Ls180	268.7 ± 7.1	531.2 ± 2.9	8.6 ± 0.5	7.7 ± 0.8	47.7 ± 1.0

Values are presented as means and SD (n=9).

Table S4. Selectivity index (SI) between IC_{50} values for healthy cells (CCD841 CoN line) and cancer cells.

No	Cell line	CE	F1	F2	F3	F4
				SI		
1	Caco-2	1.0	1.6	2.1	0.9	1.6
2	Ht-29	1.0	0.7	2.5	2.0	1.3
3	Dld-1	0.8	0.9	1.0	1.0	1.3
4	Ls180	1.1	0.8	2.5	1.4	2.2
